# Supplementary material for: Discovery of Novel Trace Amine-Associated Receptor 5 (TAAR5) Antagonists Using a Deep Convolutional Neural Network
Source: Int J Mol Sci. 2022 Mar 14;23(6):3127. doi: 10.3390/ijms23063127 (PMC8954676; doi:10.3390/ijms23063127)
Supplement: Supplementary file 1 [file ijms-23-03127-s001.zip › ijms-1606344.pdf]

Supplementary Figures

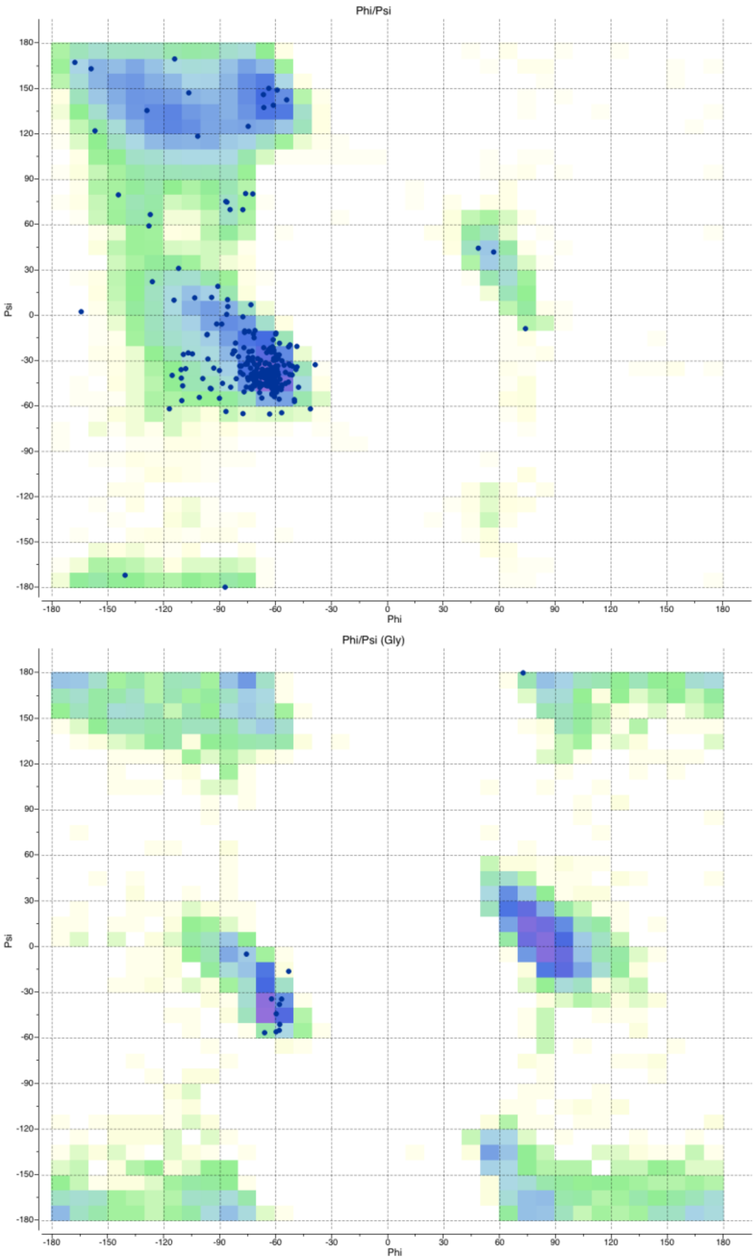

Supplementary Figure 1: Ramachandran plot. Ramachandran plots of the homology modeled mTAAR5 receptor. The dark blue regions contain the most favorable combinations of phi/psi. The light green regions indicate the allowed regions, while disfavored parameters fall out of the light yellow areas.

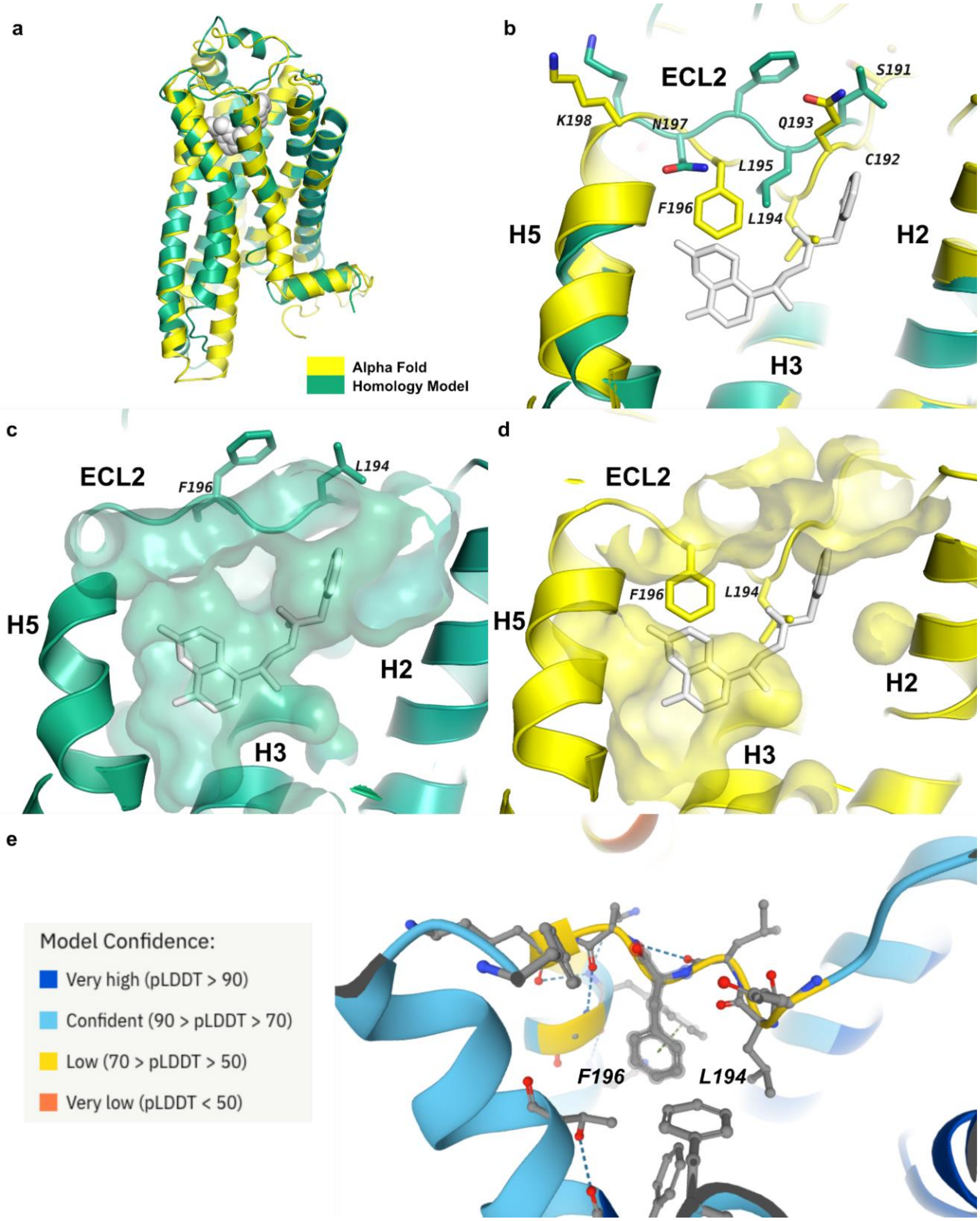

Supplementary Figure 2. Comparison of the homology model and AlphaFold predicted mTAAR5 structures. (A) An overlay of the cartoon representation of constructed mTAAR5 model and the AlphaFold predicted mTAAR5 structure with the bound ligand shown as a sphere. (B) A close-up view of the binding site. Side chains of ECL2 shown in stick and labeled. The bound ligand found in the template structure (PDBID: 6IBL) shown in white. (C) Surface representation of the binding site of mTAAR5 homology model. (D) Surface representation of binding cavity of AlphaFold predicted mTAAR5 structure around the bound ligand. (E) Residues of ECL2 of AlphaFold predicted mTAAR5 structures colored with Model Confidence (figure adopted from https://alphafold.ebi.ac.uk/).
